# Secondary resistance to immunotherapy associated with β-catenin pathway activation or PTEN loss in metastatic melanoma

**DOI:** 10.1186/s40425-019-0780-0

**Published:** 2019-11-08

**Authors:** Jonathan A. Trujillo, Jason J. Luke, Yuanyuan Zha, Jeremy P. Segal, Lauren L. Ritterhouse, Stefani Spranger, Karen Matijevich, Thomas F. Gajewski

**Affiliations:** 1 Department of Medicine, University of Chicago, 5841 S. Maryland Ave, MC2115, Chicago, IL 60637 USA; 2 Department of Pathology, University of Chicago, 5841 S. Maryland Ave, MC2115, Chicago, IL 60637 USA; 3 Present address: Koch Institute for Integrative Cancer Research at Massachusetts Institute of Technology, Department of Biology at MIT, Cambridge, USA

**Keywords:** Immune checkpoint blockade, Peptide vaccine, Secondary resistance, PTEN-loss, β-Catenin activation, Immune exclusion, Next-generation sequencing

## Abstract

**Background:**

While cancer immunotherapies including checkpoint blockade antibodies, adoptive T cell therapy, and even some vaccines have given rise to major clinical responses with durability in many cases, a subset of patients who initially respond subsequently develop secondary resistance to therapy. Tumor-intrinsic mechanisms of acquired immunotherapy resistance are incompletely understood.

**Methods:**

Baseline and treatment-resistant tumors underwent molecular analysis via transcriptional profiling or genomic sequencing for oncogenic alterations and histologic analysis for T cell infiltration to investigate mechanisms contributing to T cell exclusion and acquired resistance to immunotherapy**.**

**Results:**

We describe two patients with metastatic melanoma who initially showed a durable partial response to either a melanoma-peptide/interleukin-12 vaccine or combined anti-CTLA-4 + anti-PD-1 therapy, but subsequently developed new treatment-resistant metastases. In the first case, the recurrent tumor showed new robust tumor expression of β-catenin, whereas in the second case genomic sequencing revealed acquired PTEN loss. Both cases were associated with loss of T cell infiltration, and both pathways have been mechanistically linked to immune resistance preclinically.

**Conclusion:**

Our results suggest that secondary resistance to immunotherapies can arise upon selection for new oncogenic variants that mediate T cell exclusion. To identify the spectrum of underlying mechanisms of therapeutic resistance, similar evaluation for the emergence of tumor-intrinsic alterations in resistant lesions should be done prospectively at the time of relapse in a range of additional patients developing secondary resistance.

## Background

Approximately 22–60% of patients with metastatic melanoma who have objective responses to immunotherapies such as anti-PD-1 and/or anti-CTLA-4 antibodies will subsequently relapse [1–4]. Mechanisms of immune-resistant cancer progression in this context are incompletely understood. While a significant focus has been placed on interrogating baseline tumor biopsies for genomic and immune determinants of primary resistance, longitudinal analysis of tumors at disease progression are needed to uncover molecular drivers of secondary resistance.

Several cases of secondary resistance to immunotherapies have been reported that revealed tumor cell-intrinsic defects in antigen processing/presentation [5–9] and in IFN-γ signaling [10–12]. Early studies found evidence that melanoma patients who initially responded to cytokines and adoptive T cell-based therapies developed secondary resistance through cancer cell loss of beta-2 microglobulin (B2M), the subunit necessary for antigen presentation by MHC class I molecules [5]. Analysis of longitudinal tumor biopsy specimens from metastatic melanoma patients treated with anti-CTLA-4 or anti-PD-1 identified a subset of initial responders whose disease progressed with resistant tumors no longer expressing B2M [6]. Recently, acquired B2M loss also has been identified in a metastatic melanoma patient with secondary resistance to PD-1 blockade [7], in a case of lung cancer that developed resistance to the anti-CTLA-4 + anti-PD-1 combination [8], and in resistant brain metastases in two patients with mismatch-repair deficient colorectal cancer that acquired resistance to anti-PD-1 therapy [13]. Defective IFN-γ signaling, such as through inactivating mutations in Janus kinases (JAK1 or JAK2) or in the interferon-gamma receptor 1 (IFNGR1), has also been proposed to correlate with resistance to anti-PD-1 therapy [7, 11, 12]. Genome-scale CRISPR-Cas9 mutagenesis screens of cancer cells have provided evidence for a causal relationship between defects in antigen processing and presentation machinery in promoting resistance to T cell-based immunotherapies [14, 15]. Thus, loss of B2M and defective IFN-γ signaling can contribute to a T cell-resistant phenotype and are tumor-intrinsic determinants of resistance to immunotherapies. However, such defects are not found in all tumors, and these escape mechanisms are difficult to drug therapeutically. Thus, continued analysis of secondary resistance samples is important, in the hope of identifying pathways that might be amenable to future therapeutic intervention.

Molecular analysis of baseline tumor biopsies has revealed that selected oncogenic alterations in tumor cells can promote immune cell exclusion from the tumor microenvironment and may contribute to primary immunotherapy resistance. In pre-clinical studies, tumor cell-intrinsic activation of the Wnt/β-catenin pathway has been identified to mediate T cell exclusion from the tumor microenvironment and primary resistance to immune checkpoint blockade therapy [16]. Mechanistic studies using a genetically engineered mouse model of melanoma revealed that β-catenin activation resulted in a loss of BATF3-lineage dendritic cells in the tumor microenvironment, leading to failure of T cell priming and lack of T cell accumulation in tumors. Adoptively transferred tumor-specific T cells or prophylactic vaccination aimed at inducing endogenous anti-tumor memory CD8^+^ T cells also failed to control β-catenin-expressing tumors in this model, due to defective effector T cell trafficking [17]. These data demonstrated that tumor cell-intrinsic β-catenin activation confers an immune-resistant phenotype that impairs immune control even in the face of therapeutically induced anti-tumor T cells. These findings raise the possibility that tumor recrudescence could occur as a consequence of upregulation of β-catenin by cancer cells, resulting in secondary resistance to immunotherapy. Beyond β-catenin, gene deletions and loss-of-function mutations of the tumor suppressor phosphatase and tensin homolog (PTEN) have also been associated with poor T cell infiltration in the tumor microenvironment in metastatic melanoma [18]. Loss of PTEN, which leads to increased activation of the phosphatidylinositol 3-kinase (PI3K)-Akt pathway, has been associated with primary resistance to PD-1 blockade in melanoma [18]. Whether acquired PTEN loss leads to secondary immune resistance to immune checkpoint therapies in melanoma has not been reported.

In this context, we describe two patients who initially showed a durable partial response to immunotherapy yet subsequently developed new treatment-resistant metastases. Both cases showed loss of a T cell-inflamed tumor microenvironment, providing an opportunity to investigate potential molecular aberrations associated with loss of T cell infiltration and immunotherapy resistance.

## Methods

### Immunohistochemistry

Immunohistochemistry (IHC) for S-100, Melan-A and HMB-45 and the respective controls were performed on formalin-fixed, paraffin-embedded (FFPE) tissue sections by the Clinical Hematology and Immunohistochemistry Laboratories of the University of Chicago Hospitals. Stained IHC samples were evaluated by clinical pathologists at the University of Chicago Hospitals. CD8 and β-catenin immunohistochemistry staining was performed by the Human Tissue Resource Center (HTRC) at the University of Chicago. Immunohistochemistry staining was performed using a CD8-specific monoclonal antibody (Ab, CD8 clone C8/144B, R&D Systems) and a β-catenin monoclonal Ab (clone CAT-5H1, Life Technologies) in combination with a secondary goat anti-mouse immunoglobulin G (IgG) conjugated to an alkaline phosphatase (Biocare Medical). Slides were scanned using a CRi Panoramic Scan Whole Slide Scanner and viewed with Panoramic Viewer 1.15.4 (3DHISTECH).

### Multiplex immunofluorescence

Multiplex immunofluorescence (IF) was done according to Opal kit (Perkin Elmer) instruction. IF staining was performed using PTEN Ab (clone 6H2.1, EMD), CD8 Ab (clone C8/144B, R&D Systems) and Sox10 Ab (clone 20B7, R&D Systems). Briefly, FFPE tissue sections were baked for 1 h at 65 °C, cleared by submerging in histoclear solution (Fisher) for 10 min three times. The sections were then rehydrated by submerging in 100, 95, and 75% ethanol solutions, rinsed in distilled water, and fixed in 10% normal buffered formalin solution for 20 min. After rinsing in water, the slides were placed in EDTA (pH 9) buffer. Antigen retrieval was performed in TintoRetriever Pressure cooker at 115 °C for 20 min. The tissue sections were then blocked with proper blocking buffer, incubated with PTEN Ab for 1 h at room temperature, washed three times in Tris-buffered saline with Tween 20 (TBST) buffer (pH 7.6), incubated with HRP-conjugated secondary Ab, followed by three washes in TBST, and incubated with proper Opal reagent for 10 min at room temperature. The procedure was then repeated for CD8 and Sox10. After all the targets were labeled, the sections were incubated with DAPI solution for 5 min at room temperature, and mounted in ProLong Diamond Antifade Mountant (Invitrogen). The tissue sections were then scanned using Vectra Polaris (Perkin Elmer) and the images were captured using Phenochart (Perkin Elmer).

### Gene expression profiling

Samples were obtained from eligible patients who signed written informed consent for clinical trials and tissue biobanking at the University of Chicago. Core biopsies were obtained from material resected from patients as part of standard clinical management. Tumor was grossly isolated from surrounding normal tissue and a small piece of tumor was snap frozen in liquid nitrogen. RNA was later isolated from cryopreserved tumor biopsy using Allprep DNA/RNA mini kit (Qiagen, Inc) and quality controlled by the Human Immunologic Monitoring Facility at the University of Chicago. The transcription profiling was done using the Human Genome U133 plus 2.0 Array (Affymetrix) at the Genomic Core Facility at the University of Chicago. Subsequent data analysis involved global normalization of array values to the median signal intensity of all genes on the array. The gene expression values are log2-transformed.

### In vitro T cell priming and ELISpot

Heparinized blood was drawn before treatment, monthly on treatment, and at the end of the vaccine study. The four peptides used in the vaccine include: Melan-A (AAGIGILTV), gp100 (KTWGQYWQV), MAGE-3 (FLWGPRALV), and NA17 (VLPDVFIRCV). Peripheral blood mononuclear cells (PBMCs) were isolated using Ficoll-Hypaque gradient centrifugation and cryopreserved in liquid nitrogen freezer vapor phase. Antigen-specific CD8^+^ T cells were expanded through an in vitro stimulation step. Briefly, the PBMCs were thawed. CD8^+^ cells were isolated using CD8 microbeads (Miltenyi Biotech). The flow through CD8-negative cells were pulsed with 50 μM peptide (either derived from Epstein-Barr virus [EBV; GLCTLVAML), Melan-A (AAGIGILTV), gp100 (KTWGQYWQV), MAGE-3 (FLWGPRALV), or NA17 (VLPDVFIRCV)] in the presence of 2.5 μg/ml beta-2 microglobulin for 1 h at 37 °C. The peptide pulsed CD8-negative cells were then washed and irradiated with a total dose of 3000 rad and co-culture with CD8^+^ cells at a 5:1 ratio for 5 days at 37 °C. Recombinant human IL-2 (rhIL-2) at a concentration of 20 units/ml was added into the culture on day 2. On day 5, CD8^+^ cells were collected and co-cultured with irradiated, peptide pulsed CD8-negative cells and rhIL-2 for another 5 days. On day 10, expanded CD8^+^ cells were collected and seeded on the ELISpot plate pre-coated with IFN-γ Ab (clone 1-D1K, Mabtech, Inc) and co-cultured with peptide-pulsed T2 cells overnight. On the following day, the plate was washed and incubated with a biotinylated anti-IFN-γ secondary Ab (clone 7-B6–1, Mabtech, Inc.) for 2 h at room temperature. Following three washes, the plate was incubated with streptavidin-conjugated AP for 1 h, washed, and incubated with AP substrate. Excess substrate was removed by rinsing with tap water. The plate was then air-dried, captured, and counted using a CTL-ImmunoSpot S6 Core Analyzer (Cellular Technology Ltd). All samples were analyzed in triplicate.

### Next-generation genomic sequencing

Next-generation genomic sequencing (NGS) was performed using the OncoScreen ST2.0 or OncoPlus, the University of Chicago Clinical Laboratory Improvement Amendments-certified next-generation sequencing platforms [19]. The OncoScreen ST2.0 clinical assay was performed on tissue derived from the wide local excision scalp melanoma. OncoSreen ST2.0 is a 50-gene solid tumor panel that uses the Ion Ampliseq Cancer Hotspot Panel V2 primer set (Thermo Fisher Scientific) for amplification of 207 hot-spot targeted amplicons across 50 genes [19]. The OncoScreen ST2.0 platform includes the genes listed in Additional file 1: Table S1.

Tissue slides and blocks were reviewed by a pathologist to select the appropriate material for NGS testing. DNA was isolated from micro-dissected FFPE tumor tissue using the QIAamp DNA FFPE Tissue Kit (Qiagen). Following extraction, DNA was quantified using the Qubit fluorometric assay (Thermo Fisher Scientific) and further assessed for quantity and quality using a quantitative PCR assay (hgDNA Quantitation and QC kit, KAPA Biosystems). FFPE DNA was amplified for somatic mutations located within mutational hotspot regions of 50 cancer-related genes using multiplex PCR reagents (Thermo Fisher Scientific). PCR products were quantitated using the Qubit assay then used as a substrate for NGS library preparation (HTP Library Preparation Kit, KAPA Biosystems), using selected patient-specific adapter index sequences. Libraries were quantified using a quantitative PCR assay (Library Quantification Kit, KAPA Biosystems), then pooled and sequenced via the Illumina MiSeq system (2 × 152 bp paired-end sequencing). Sequencing data was analyzed via custom-designed bioinformatics pipelines on an University of Chicago HIPAA compliant high performance computing system, using the hg19 (GRCh37) human genome reference sequence for alignment [19]. Limit of detection: 5% mutant alleles.

The resistant cerebellar metastases underwent next-generation genomic sequencing using the OncoPlus assay, a clinically validated hybrid capture genomic sequencing platform comprising 1212 commonly altered cancer genes for mutational and copy number analysis (genes listed in Additional file 1: Table S2) [19].

A pathologist reviewed the original pathology report, examined candidate H&E stained slides, and selected the appropriate block for NGS testing. DNA was isolated from microdissected FFPE tumor tissue using the QiaAMP DNA FFPE Tissue Kit (Qiagen). Following extraction, DNA was quantified using the Qubit fluorometric assay (Thermo Fisher Scientific) and further assessed for quantity and quality using a quantitative PCR assay (hgDNA Quantitation and QC kit, KAPA Biosystems). DNA was subjected to ultrasonic fragmentation and subsequent library preparation using adapter molecules containing patient-specific index sequences (HTP LibraryPreparation Kit, Kapa Biosystems). After library amplification, quantification and pooling, fragments originating from targeted genomic regions were enriched using a panel of biotinylated oligonucleotides (SeqCap EZ, Roche Nimblegen) supplemented with additional oligonucleotides (xGen Lockdown Probes, IDT). After subsequent amplification and pooled library quantification, libraries were sequenced in rapid run mode on a HiSeq 2500 system (Illumina) to produce 2 × 101 bp paired end sequencing reads. Sequencing data was analyzed via custom-designed bioinformatics pipelines on an University of Chicago HIPAA compliant high performance computing system, using the hg19 (GRCh37) human genome reference sequence for alignment. Limit of detection: For mutations, insertions and deletions, limit of detection is 10% mutant alleles (roughly corresponding to 20% tumor cells). Limit of detection for fusions/translocations is 20% tumor cells. Gene fusions cannot be detected in the rare occurrence of a fusion between ALK, RET or ROS1 and a partner gene less than 100,000 bp distant. Limit of detection for copy number changes is >4X or < 0.5X normal copy number, with relevant equivocal changes reported at >2X or < 0.6X.

## Results

### Secondary immune resistance associated with β-catenin activation

A 54-year-old Caucasian male with metastatic melanoma (diagnosed prior to the era of B-Raf inhibitors) initially received interleukin-2 without benefit followed by right hepatic lobectomy. Two years following surgery pulmonary metastases were observed leading to mediastinoscopy with biopsy of right level 4, lower paratracheal lymph node. Pathology was consistent with metastatic melanoma with tumor cells extensively immunoreactive for HMB-45 and focally immunoreactive for Melan-A and S-100. The patient was HLA-A2-positive and enrolled on a clinical trial of a multi-peptide vaccine combined with interleukin-12 [20]. The patient was treated every 3 weeks for one year and a durable partial response was observed (RECIST 1.0) [20]. The patient was followed by close observation by serial computed tomography scans until a new metastatic lesion in the pelvis was confirmed by biopsy approximately 3 years later.

**Fig. 1:**
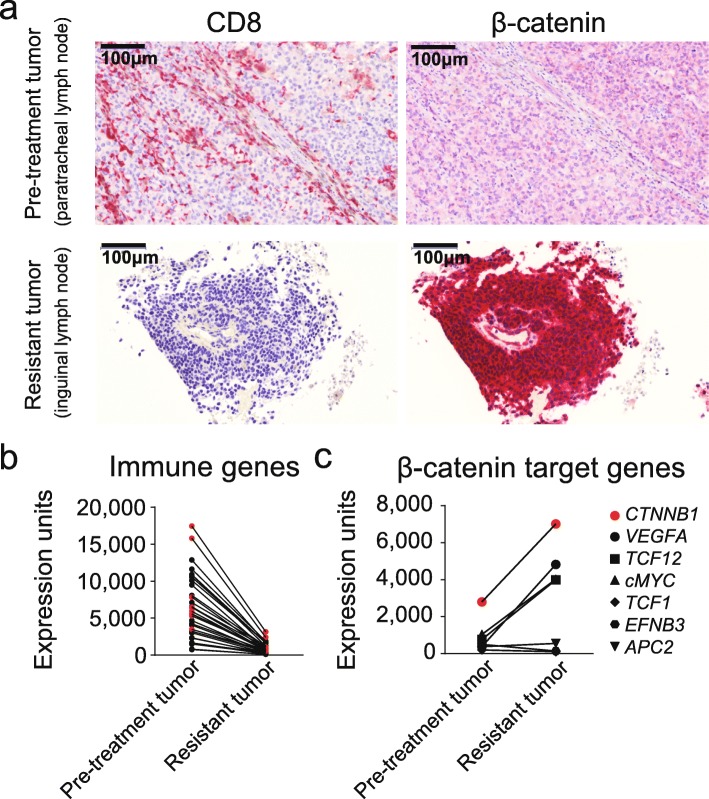
Tumor gene expression profiling, CD8^+^ T cell infiltration, and β-catenin status at baseline and at recurrence. **a** Immunohistochemistry staining for CD8 (red staining) and β-catenin (red staining), in baseline (pre-treatment, right lower paratracheal lymph node metastasis) and recurrent (treatment-resistant, left inguinal lymph node metastasis) melanoma tumor biopsies. **b** Expression level of immune-related genes in baseline and recurrent tumor samples measured by genome expression microarray. Depicted are the genes *GZMK, CD8A, CCL4, CXCL9, CCL3, CCL5, HLADMA, CXCL10, TRGC2, TRAA, NKG7, CD2, TRGV9, TRGC2, PRF1, CD8B, TRBC1, CD38, IL1R2, IL23A, TRBC1, IL2RG, CCL18, CD27, IFNG, RAC2, TNFSF10, CD3E, TAP1, TNFRSF9, HLADPA1, TAP2, NLRP1, STAT1, CXCL13*. Genes in bold font are being shown in red and were previously part of our core signature associated with CD8^+^ T cells [21]. **c** Gene expression levels of six β-catenin target genes (*VEGFA, TCF12, MYC, TCF1, EFNB3, APC2*) as well as β-catenin (*CTNNB1*, red) itself. Genome microarray data (b and c): expression levels for each gene transcript are normalized to median signal intensity of all genes on the microarray, and represented as normalized hybridization intensity data and expressed as expression units

**Fig. 2:**
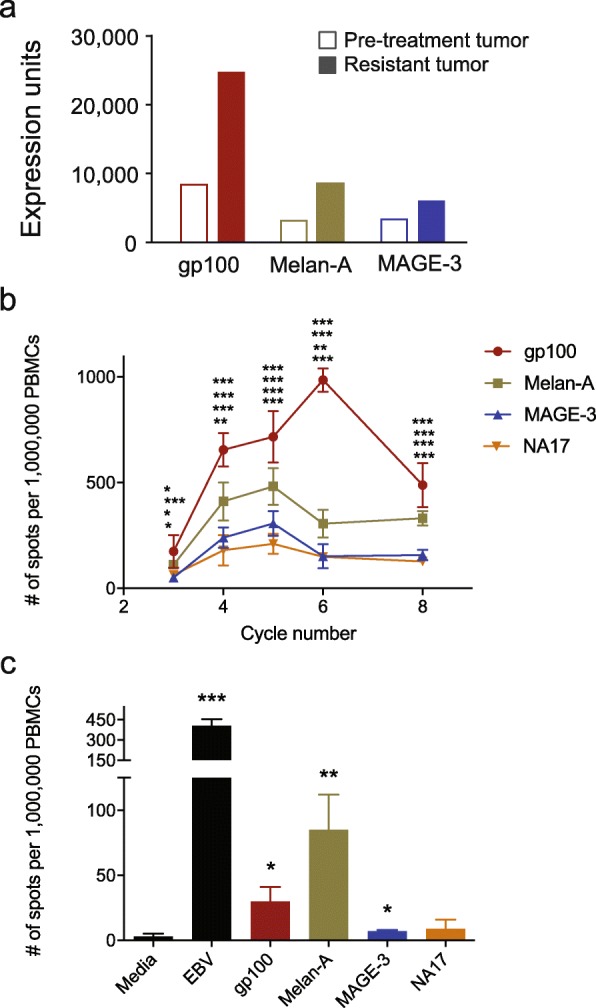
Peripheral tumor-reactive T cells persist at the time of progression. **a** Expression-level of targeted tumor antigens in pre-treatment (unfilled bars) and resistant (filled bars) tumor samples determined by genome expression microarray (NA-17 not represented on the gene array). Gene expression of targeted antigen transcripts are normalized to median signal intensity of all genes on the array and expressed as expression units. **b** IFN-γ ELISpot assessing the T cell reactivity against the four peptides used in the vaccine, gp100, Melan-A, MAGE-3, NA-17, over time during initial treatment. PBMCs isolated at each time point and stimulated with the indicated melanoma peptides or media control. Samples analyzed in triplicate and presented as the mean number of spots per number of PBMCs with standard deviation. Mean number of spots for each peptide compared to media control. *P*-values for gp100, Melan-A, MAGE-3, and NA17 peptide versus media control listed from top to bottom, respectively, at each time point, *p < 0.05, **p < 0.001, ***p < 000.1 (**c**) IFN-γ ELISpot showing persistent T cell reactivity against three melanoma peptides (gp100, Melan-A and MAGE-3) at the time of progression. PBMCs stimulated with media control, EBV antigen (control peptide), gp100, Melan-A, MAGE-3, and NA17 peptide. Samples analyzed in triplicate and presented as the mean number of spots per number of PBMCs with standard deviation. Mean number of spots compared to media control. *p < 0.05, **p < 0.001, ***p < 000.1

A biopsy was performed of the new lesion, both to confirm recurrent melanoma and to study the immunobiology of the tumor microenvironment relative to that of the pre-treatment tumor. Immunohistochemical studies showed extensive staining by HMB-45 and focal immunoreactivity for Melan-A and S100, confirming melanoma and expression of these two antigens in the new lesion. Analysis of the pretreatment biopsy revealed strong and homogenous CD8^+^ T cell infiltration (Fig. 1a upper left panel). Consistent with immunohistochemistry analysis, gene expression profiling revealed evidence for a T cell-inflamed tumor microenvironment including T cell markers, chemokines, and interferon-induced genes (Fig. 1b). In contrast, immunohistochemical staining of the recurrent tumor showed absence of infiltrating CD8^+^ T cells (Fig. 1a lower left panel). Gene expression profiling revealed markedly reduced chemokines and other immune genes compared to the original tumor biopsy (Fig. 1b), consistent with selection for a microenvironment that failed to recruit T cells. Based on the ability of activated β-catenin to mediate T cell exclusion [16], stabilized β-catenin was analyzed by immunohistochemistry. Strikingly, the pre-treatment sample had minimal staining for β-catenin, whereas the recurrent tumor showed strong staining that included nuclear localization (Fig. 1a right panels). Expression of four defined β-catenin target genes and also of β-catenin transcripts were upregulated in the recurrent tumor (Fig. 1c). Thus, the immune resistance phenotype exhibited by the new metastases was associated with β-catenin pathway activation. Expression of three of the four antigens targeted by the vaccine (Melan-A, MAGE-3, gp100) was detected in the pre-treatment tumor specimen by gene-expression microarray analysis (Fig. 2a). Retained expression of tumor antigens targeted by the vaccine was assessed by gene-expression microarray analysis, and Melan-A, MAGE-3, and gp100 were all confirmed to be expressed by the recurrent tumor (Fig. 2a). Analysis of peripheral blood indicated an increase in T cell reactivity against all four peptides used in the vaccine, Melan-A (AAGIGILTV), gp100 (KTWGQYWQV), MAGE-3 (FLWGPRALV), and NA-17 (VLPDVFIRCV) during initial treatment (Fig. 2b). Re-analysis of the T cell responses from peripheral blood obtained at the time of progression revealed persistent reactivity against three of the peptides (gp100, Melan-A and MAGE-3), consistent with T cell memory against at least these three epitopes (Fig. 2c). The patient was subsequently treated with dacarbazine chemotherapy, which resulted in a partial response.

### Secondary immune resistance associated with biallelic PTEN loss

A 23-year-old Asian male with metastatic BRAF-V600E melanoma was originally treated with B-Raf inhibitor + MEK inhibitor (trametinib and dabrafenib) and palliative radiation to a sacral metastasis. The patient had a mixed response to therapy, and was subsequently treated with combination anti-CTLA-4 + anti-PD-1 therapy with ipilimumab and nivolumab according to the FDA-approved dose and schedule. The patient achieved a durable partial response to therapy. Eight months later, the patient developed a left midclavicular nodule that was biopsied and confirmed to be metastatic melanoma and subsequently treated with radiation. The patient continued therapy with nivolumab for a total of fourteen months until imaging demonstrated early evidence of disease progression prompting reinduction with ipilimumab + nivolumab. After eighteen months total on immune checkpoint blockade, the patient developed multi-site disease progression, including new osseous lesions, mediastinal and hilar lymphadenopathy, and a cerebellar tumor. The patient underwent a craniotomy and resection of the cerebellar tumor that confirmed metastatic melanoma. The patient ultimately received palliative radiation and ultimately died with progressive metastatic disease. To explore mechanisms of immunotherapy resistance, pre-treatment and treatment-resistant tumor biopsies were analyzed for somatic genetic abnormalities.

**Fig. 3:**
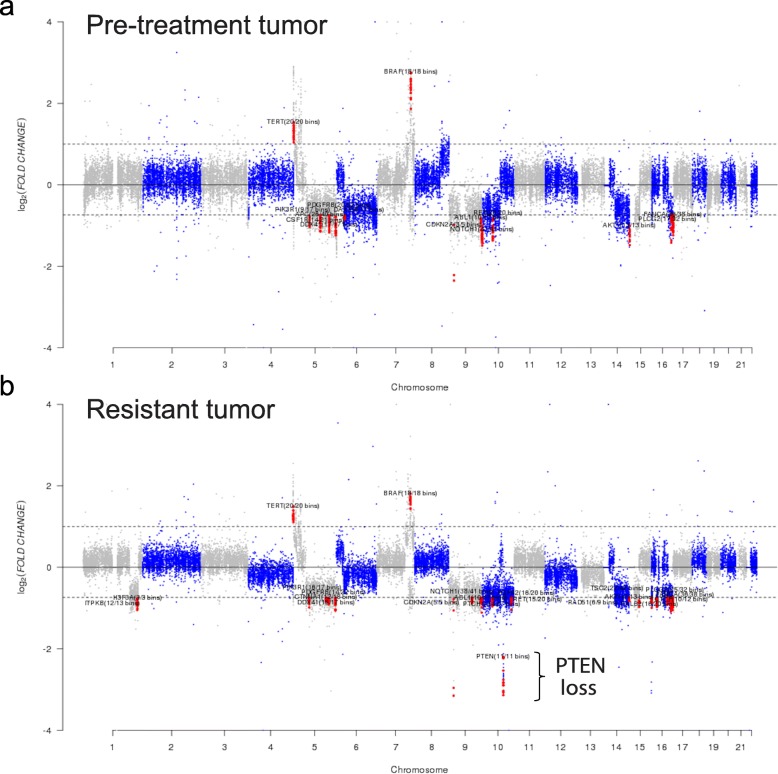
Acquired genetic loss of PTEN in a therapy-resistant melanoma tumor in a patient previously responding to ant-CTLA-4 and anti-PD-1 therapy. **a** and **b** Copy number alterations. Next generation sequencing of pre-treatment and therapeutic-resistant melanoma lesions shows acquired loss of PTEN in the treatment resistant tumor specimen but not the pre-treatment lesion. Log2 of fold- changes in the (**a**) pre-treatment tumor sample (upper panel) and (**b**) treatment-resistant metastasis (lower panel). The analysis shows copy number changes in *BRAF, PTEN, CDKN2A, FANCA, H3F3A, NOTCH1, PALB2, RAD51, RET, TSC1, TSC2*. Copy number alterations are indicated in red. Genomic regions across the chromosomes that have no detectable alterations are indicated in blue or gray. Dotted lines indicate the Log2 fold-change cutoffs

Tumors were analyzed by next-generation genomic sequencing (NGS) using a clinically validated amplicon-based assay (OncoScreen ST2.0) or a hybrid capture genomic sequencing platform (OncoPlus), respectively, comprising a panel of commonly altered cancer genes for mutational and copy number analysis (Fig. 3 and Table 1). The pathogenic variants detected in the pre-treatment scalp melanoma included the BRAF-V600E mutation (BRAF c.1799 T > A, p.V600E), amplification of BRAF located on chromosome 7q34, and loss of the tumor suppressor gene CDKN2A located on chromosome 9p21.3 (Fig. 3a). The treatment-resistant cerebellar metastasis also had the same BRAF-V600E mutation (BRAF c.1799 T > A, p.V600E), loss of CDKN2A, and BRAF amplification, but additionally demonstrated biallelic loss of the tumor suppressor gene PTEN located on chromosome 10q23.31 (Fig. 3b). Both the pre-treatment and resistant tumors shared the BRAF amplification, which has been suggested to confer relative resistance to BRAF inhibitor treatment [22]. Loss of CDKN2A has been suggested to cooperate with PTEN deletion to drive resistance to BRAF inhibitors [23]. The treatment-resistant metastasis uniquely harbored biallelic PTEN loss, while the pre-treatment biopsy had no detectable PTEN alterations. No mutations were observed in the gene encoding beta-2-microglobulin (B2M), the required subunit necessary for surface expression of the MHC class I molecule, or the gene encoding interferon-receptor-associated Janus kinase 2 (JAK2) in either of the tumor specimens. No mutations conferring microsatellite instability were observed in the pre-treatment or treatment-resistant tumors. Additional somatic alterations and copy-number events (Table 1) identified in the treatment-resistant tumor were of uncertain significance.

**Table 1:** Genetic variants detected via next-generation sequencing of pre-treatment and treatment-resistant tumor specimens

Baseline (pre-treatment) biopsy	Treatment-resistant biopsy
Variants detected (NGS platform: OncoScreen ST2.0)	Variants detected (NGS platform: OncoPlus)
*Pathogenic variants*	*Pathogenic variants*
BRAF c.1799 T > A, p.V600E	BRAF c.1799 T > A, p.V600E
CDKN2A loss	CDKN2A loss
BRAF amplification	BRAF amplification
	PTEN loss
	*Variants of indeterminate clinical significance*
	APC c.385G > C, p.E129Q
	APC c.6211A > G, p.I2071V
	PTCH1 c.3641C > T, p.T1214 M
	RET c.2939 + 6C > T
	FANCA Loss
	H3F3A Loss
	NOTCH1 Loss
	PALB2 Loss Equivocal
	RAD51 Loss Equivocal
	RET Loss
	TSC1 Loss Equivocal
	TSC2 Loss Equivocal

Abbreviations: *NGS* next-generation genomic sequencing, *c*. cDNA alteration, *p*., protein alteration

**Fig. 4:**
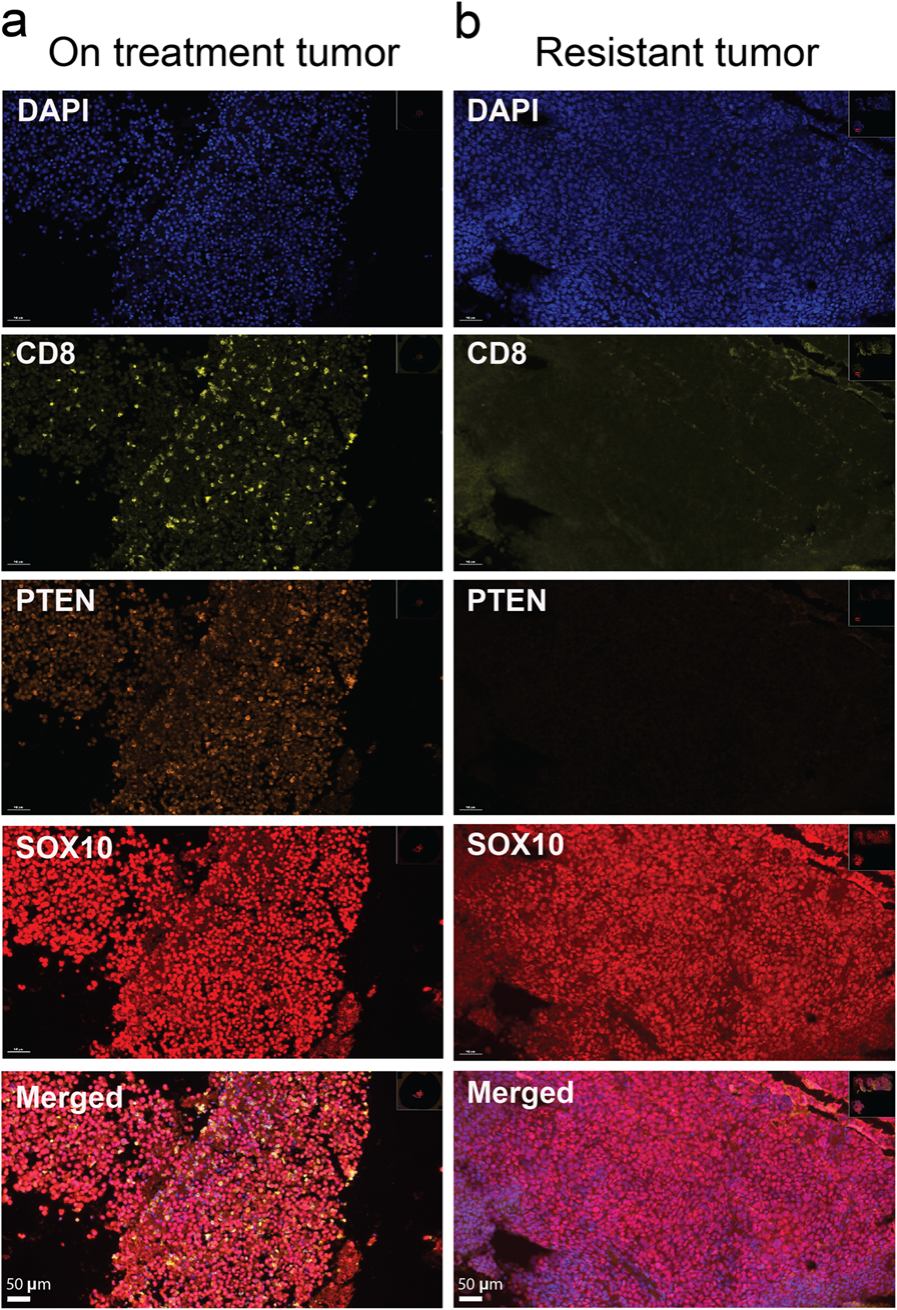
Loss of PTEN protein expression by melanoma cells associated with a lack of CD8^+^ T cell infiltration. **a** Immunofluorescence demonstrates that the on-treatment specimen shows PTEN protein expression by SOX10-positive melanoma cells and CD8^+^ T cell infiltration (left panels); (**b**) the therapeutic-resistant post-treatment specimen (right panels) from the same patient shows minimal PTEN protein expression by SOX10-positive melanoma cells and no CD8^+^ T cell infiltration. Multiplex immunofluorescence staining was performed for DAPI, Sox10, CD8, and PTEN; each stain shown separately and merged

To determine whether genetic alterations in PTEN led to loss of protein expression, multiplex immunofluorescence was performed on the on-treatment midclavicular tumor biopsy during disease control and on the treatment-resistant cerebellar tumor biopsy (Fig. 4). Histologic analysis of the biopsies revealed extensive expression of Sox10 identifying melanoma tumor tissue. While PTEN protein was expressed throughout the first biopsy during tumor control, it was absent from the second lesion that represented disease progression following nivolumab + ipilimumab. The treatment-resistant biopsy also revealed minimal staining for CD8^+^ T cells compared to the earlier biopsy (Fig. 4), and was additionally associated with loss of stainable PTEN protein. These results were noteworthy based on prior mechanistic data indicating immunotherapy resistance upon PTEN loss [18].

## Discussion

Immunotherapeutic interventions, including checkpoint blockade, adoptive T cell transfer, and some vaccination approaches have been developed as potent strategies to induce and enhance anti-tumor immunity, translating into clinical efficacy in multiple tumor types [24]. Translational studies of anti-PD-1 antibodies and also experimental vaccines have provided evidence that clinical response is favored when CD8^+^ T cells are present within the tumor microenvironment at baseline [25]. Absence of recruitment and activation of tumor antigen-specific T cells in the tumor microenvironment has therefore been correlated with primary resistance to anti-PD-1 immunotherapy [26]. Two oncogenic events linked to poor T cell infiltration and primary immunotherapy resistance are tumor cell-intrinsic β-catenin pathway activation and also PTEN loss-of-function mutation or deletion [16, 18]. However, whether secondary resistance might arise through acquisition of tumor cell-intrinsic oncogenic alterations had not been known. Our current data provide evidence that acquisition of active β-catenin signaling in tumor cells or PTEN loss might mediate secondary resistance to immunotherapy even in the presence of circulating memory CD8^+^ T cells specific for tumor-expressed antigens.

Our results suggest that a broad net should be cast when evaluating for mechanisms of secondary resistance to immunotherapy in patients. Recent data have revealed that loss-of-function defects in beta-2 microglobulin and also Jak signaling can be found in tumors that progress following initial clinical response to anti-PD-1 [7]. Our current results argue that active immune exclusion mechanisms of resistance also can occur, as in the β-catenin protein stabilization identified in the vaccine-treated patient. There was not sufficient tissue obtained in the recurrent tumor biopsy for exome or genomic sequencing to elucidate the mechanism of β-catenin activation in this case, but our previous results have demonstrated that β-catenin pathway activation in melanoma can be driven by activating mutations in CTNNB1 (β-catenin) itself, inactivating mutations in inhibitors of β-catenin such as AXIN1, or over-expression of specific Wnt ligands or Frizzled receptors [16]. Alterations that lead to Wnt/β-catenin pathway activation are recurrent in melanoma [16, 27] and other tumor types [28] and are associated with a lack of T cell infiltration at baseline; however, clinical immunotherapy-specific outcome data, especially for immune-checkpoint inhibitors, are still lacking. The current patient developed a β-catenin-expressing tumor variant associated with immune escape. Immune surveillance and long-term protection against re-emerging cancer cells depends upon retention of tumor antigens and the presence of tumor-specific T cells. In this case, acquired immune resistance was not associated with loss of expression of melanoma antigens by the new metastasis nor linked to an absence of melanoma-specific T cells from the immune repertoire. Rather, T cells failed to accumulate in the new resistant tumor in spite of the presence of circulating memory T cells specific to three of the melanoma epitopes targeted by the peptide vaccine. It is of interest that he subsequently responded to chemotherapy, which suggests that the mechanisms of resistance with immunotherapy versus chemotherapy may be distinct.

The patient who developed therapeutic resistance to the anti-CTLA-4 + anti-PD-1 combination progressed with multisite disease including an immune-resistant brain metastasis. The near complete absence of CD8^+^ T cells from the resected brain tumor lesion supports immune exclusion as the putative resistance mechanism, and PTEN loss may have contributed to ineffective CD8^+^ T cell accumulation. While the blood-brain-barrier regulates T cell trafficking into central nervous system tissue, it does not appear to be a major determinant of therapeutic resistance to immune checkpoint inhibitors based on the high rate of efficacy observed against melanoma metastatic to the brain [29, 30]. For example, intracranial responses to brain metastases have been observed in 57% of patients, including a 26% complete response rate to previously untreated intracranial lesions in melanoma patients treated with combined nivolumab and ipilimumab [29]. Loss of PTEN expression has been correlated with shorter time to brain metastases and reduced overall survival among patients with BRAFV600-mutant melanoma implicating the PI3K-AKT pathway in the establishment of brain metastasis [31]. Thus, the functional interaction between mutated BRAF and PTEN loss/PI3K-AKT activation in the current patient may have promoted brain metastasis and immunotherapy resistance. Concurrent biopsies of extracranial metastases were not clinically indicated and thus not performed in this patient, so we cannot rule out that distinct mechanisms apart from PTEN loss might be linked to resistant metastases in other anatomic sites. An analysis of the melanoma Cancer Genome Atlas (TCGA) data set found that the frequency of deletions and loss-of-function mutations in PTEN were greater in non-T-cell inflamed tumors [18]. In addition, the absence of PTEN protein in tumor samples correlated with diminished CD8^+^ T cell infiltration and inferior outcomes to anti-PD1 in melanoma patients [18]. While an elevated frequency of PTEN alterations has been specifically noted in melanoma brain metastases [31], combination checkpoint blockade can generate a high response rate in brain metastases [29, 30], arguing that it remains immunotherapy responsive in a major subset of cases. A prior study had reported that PTEN alterations were not correlated with an immune gene signature in brain metastases, although this analysis was not done in conjunction with clinical response [32]. Consistent with our results, biallelic loss of PTEN was exclusively identified in a treatment-resistant extracranial metastasis from a patient with metastatic uterine sarcoma who achieved a durable complete remission with anti-PD-1 therapy following resection of the sole immune-escape tumor [33].

The possibility of activation of specific oncogene pathways in immunotherapy-resistant tumors raises the potential for developing pharmacologic inhibitors of such pathways towards restoration of T cell infiltration and immunotherapy efficacy. There is renewed interest in developing inhibitors of Wnt/β-catenin signaling that might be more selective for the immune regulatory functions of this pathway. In addition, because PTEN loss-of-function results in activation of PI3 kinase, PI3K inhibitors are an attractive option to consider for potentiation of immunotherapy in PTEN-mutant cancers. Because PI3 kinase is also important for T cell activation, and in fact this represents a major signaling pathway regulated by CTLA-4 and PD-1, careful drug selection and intermittent scheduling are important considerations [34]. A pan-PI3K inhibitor was shown to block T cell activation in vivo, whereas a β-isoform-specific inhibitor was shown to improve cancer immunotherapy efficacy in a mouse model [18].

The present study has notable limitations. It describes results with only two patients, and thus additional studies involving a greater sample size will be required to determine the frequency of active β-catenin signaling or PTEN-deletion in tumor cells among cases of secondary immune resistance. Additionally, due to limited availability of biopsy tissue at each time point, not all assays (gene expression profiling, multiplex immunofluorescence, genomic sequencing) could be performed on all samples for each patient. Nevertheless, this study provides provocative examples of secondary resistance linked to loss of a T cell-inflamed tumor microenvironment.

## Conclusion

We report two cases of secondary immune resistance in metastatic melanoma patients, associated with tumor cell acquisition of either active β-catenin signaling or PTEN gene deletion, two oncogenic aberrations linked to ineffective T cell infiltration into tumor sites. Our results suggest that acquired alterations in oncogenic signaling can be added to the list of mechanisms leading to tumor outgrowth in the face of immune selective pressure catalyzed by immunotherapeutic interventions. As the number of patients treated with checkpoint inhibitors and other immunotherapies continues to grow, and as follow-up time continues to increase, it is likely that numerous additional secondary resistance cases will be identified. Such patients should be interrogated from multiple perspectives for novel mechanisms of immune escape. As these mechanisms continue to be catalogued, it is hoped that patterns will emerge and new therapies can be developed to overcome resistance clinically.

## Supplementary information

**Additional file 1: Table S1.** Genes analyzed by the OncoScreen ST2.0 next-generation genomic sequencing assay. **Table S2.** Genes analyzed by the OncoPlus next-generation genomic sequencing assay.

## Data Availability

The data sets generated and analyzed during the current study available from the corresponding author on reasonable request.

## Abbreviations

Ab: Antibody
B2M: Beta-2-microglobulin
BATF3: Basic leucine zipper ATF-like transcription factor 3
CRISPR: Clustered regularly interspaced short palindromic repeats
CTLA-4: Cytotoxic T-lymphocyte-associated protein-4
FFPE: formalin-fixed, paraffin-embedded
H&E: Hematoxylin and eosin
HIPAA: Health Insurance Portability and Accountability Act
HLA-A2: Human leukocyte Antigen-A2
HTRC: Human Tissue Resource Center
IF: Immunofluorescence
IFNGR1: Interferon-gamma receptor 1
IFN-γ: Interferon-gamma
IgG: Immunoglobulin G
IHC: Immunohistochemistry
MHC: Major histocompatibility complex
NGS: Next-generation genomic sequencing
PBMC: Peripheral blood mononuclear cells
PD-1: Programmed cell death protein-1
PI3K: Phosphatidylinositol 3-kinase
PTEN: Phosphatase and tensin homolog
RECIST 1.0: Response Evaluation Criteria in Solid Tumors guideline version 1.0
rhIL-2: Recombinant human interleukin-2
TBST: Tris-buffered saline with Tween 20
TCGA: The Cancer Genome Atlas
