# Supplementary material for: Secondary resistance to immunotherapy associated with β-catenin pathway activation or PTEN loss in metastatic melanoma
Source: J Immunother Cancer. 2019 Nov 8;7:295. doi: 10.1186/s40425-019-0780-0 (PMC6839232; doi:10.1186/s40425-019-0780-0)
Supplement: Supplementary file 1 — Additional file 1: Table S1. Genes analyzed by the OncoScreen ST2.0 next-generation genomic sequencing assay. Table S2. Genes analyzed by the OncoPlus next-generation genomic sequencing assay. [file 40425_2019_780_MOESM1_ESM.docx]

**Table S1: Genes analyzed by the OncoScreen ST2.0 next-generation genomic sequencing assay.**

| **NGS platform: OncoScreen ST2.0** | |
| --- | --- |
| **Genes analyzed for genetic alterations** | ABL1, AKT1, ALK, APC, ATM, BRAF, CDH1, CDKN2A, CSF1R, CTNNB1, EGFR, ERBB2, ERBB4, EZH2, FBXW7, FGFR1, FGFR2, FGFR3, FLT3, GNA11, GNAQ, GNAS, HNF1A, HRAS, IDH1, IDH2, JAK2, JAK3, KDR, KIT, KRAS, MET, MLH1, MPL, NOTCH1, NPM1, NRAS, PDGFRA, PIK3CA, PTEN, PTPN11, RB1, RET, SMAD4, SMARCB1, SMO, SRC, STK11, TP53, VHL |

**Table S2: Genes analyzed by the OncoPlus next-generation genomic sequencing assay.**

| **NGS platform: OncoPlus** | |
| --- | --- |
| **Gene analyzed for mutations and insertions/deletions** | ABL1, AKT1, ALK, APC, ARID1A, ARID2, ASXL1, ATM, ATR, ATRX, AXL, B2M, BAP1, BCOR, BCORL1, BIRC3, BLM, BRAF, BRCA1, BRCA2, BTK, CALR, CBL, CBLB, CCND1, CCND2, CCND3, CDH1, CDKN2A, CEBPA, CHEK1, CHEK2, CSF1R, CSF3R, CTCF, CTNNA1, CTNNB1, CUX1, CXCR4, DAXX, DDR2, DDX3X, DDX41, DICER1, DNMT3A, EGFR, EP300, EPHA3, EPHA5, ERBB2, ERBB3, ERBB4, ERCC3, ESR1, ETV6, EZH2, FANCA, FAT3, FBXW7, FGFR1, FGFR2, FGFR3, FH, FLT3, FOXL2, GATA1, GATA2, GNA11, GNAQ, GNAS, GRIN2A, H3F3A, HIST1H3B, HIST1H3C, HNF1A, HRAS, IDH1, IDH2, IKZF1, ITPKB, JAK2, KDM6A, KDR, KIT, KMT2A, KRAS, MAP2K1, MAPK1, MET, MLH1, MLH3, MPL, MRE11A, MSH2, MSH6, MTOR, MYD88, NBN, NF1, NF2, NFE2L2, NOTCH1, NOTCH2, NPM1, NRAS, PALB2, PBRM1, PDGFRA, PDGFRB, PHF6, PIK3CA, PIK3CB, PIK3R1, PLCG2, POLE, POT1, PPP2R1A, PTCH1, PTEN, PTPN11, RAD21, RAD51, RB1, RET, RUNX1, SDHB, SDHC, SDHD, SETBP1, SF3B1, SMAD4, SMARCB1, SMC1A, SMC3, SMO, SRSF2, STAG2, STK11, TERT (promoter only), TET2, TP53, TSC1, TSC2, U2AF1, VHL, WT1, ZRSR2 |
| **Genes analyzed for copy number variations** | ABL1, AKT1, ALK, APC, ARID1A, ARID2, ASXL1, ATM, ATR, AXL, B2M, BAP1, BIRC3, BLM, BRAF, BRCA1, BRCA2, CALR, CBL, CBLB, CCND1, CCND2, CCND3, CDH1, CDKN2A, CEBPA, CHEK1, CHEK2, CSF1R, CSF3R, CTCF, CTNNA1, CTNNB1, CUX1, CXCR4, DAXX, DDR2, DDX41, DICER1, DNMT3A, EGFR, EP300, EPHA3, EPHA5, ERBB2, ERBB3, ERBB4, ERCC3, ESR1, ETV6, EZH2, FANCA, FAT3, FBXW7, FGFR1, FGFR2, FGFR3, FH, FLT3, FOXL2, GATA2, GNA11, GNAQ, GNAS, GRIN2A, H3F3A, HIST1H3B, HIST1H3C, HNF1A, HRAS, IDH1, IDH2, IKZF1, ITPKB, JAK2, KDR, KIT, KMT2A, KRAS, MAP2K1, MAPK1, MET, MLH1, MLH3, MPL, MRE11A, MSH2, MSH6, MTOR, MYD88, NBN, NF1, NF2, NFE2L2, NOTCH1, NOTCH2, NPM1, NRAS, PALB2, PBRM1, PDGFRA, PDGFRB, PIK3CA, PIK3CB, PIK3R1, PLCG2, POLE, POT1, PPP2R1A, PTCH1, PTEN, PTPN11, RAD21, RAD51, RB1, RET, RUNX1, SDHB, SDHC, SDHD, SETBP1, SF3B1, SMAD4, SMARCB1, SMC3, SMO, SRSF2, STK11, TERT, TET2, TP53, TSC1, TSC2, U2AF1, VHL, WT1 |
| **Genes analyzed for fusions/translocations** | ALK, RET, ROS1 |
